# Supplementary material for: Netcombin: An algorithm for constructing optimal phylogenetic network from rooted triplets
Source: PLoS One. 2020 Sep 18;15(9):e0227842. doi: 10.1371/journal.pone.0227842 (PMC7500971; doi:10.1371/journal.pone.0227842)
Supplement: S4 File — (ZIP) [file pone.0227842.s004.zip › SequenceData-Triplet/sequenceData-10-20-30-0-Triplet/length 300, numseq 30, rate 10^-7/1 seq.pdf]

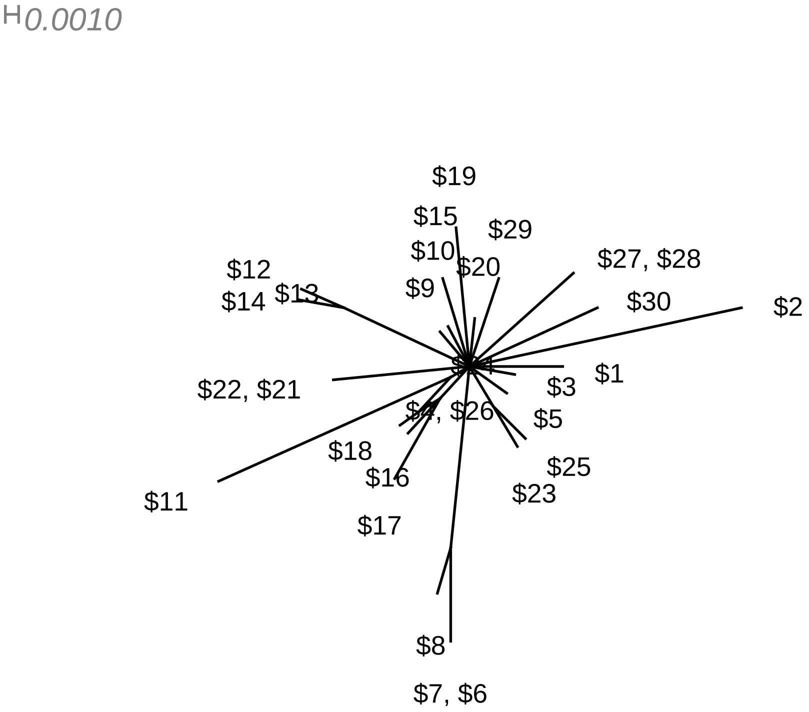
0.0010
$19
$15
$29
$10
$20
$27, $28
$12
$9
$13
$14
$30
$2
$1
$22, $21
$3
$4, $26
$5
$18
$25
$16
$23
$11
$17
$8
$7, $6
